# Modified Manikin for Tracheoinnominate Artery Fistula

**DOI:** 10.21980/J8Z93H

**Published:** 2021-07-15

**Authors:** Emily M Tarver, Gina D Jefferson, Patrick Parker, Kristina Readman, Susana M Salazar Marocho, Anna A Lerant

**Affiliations:** * University of Mississippi Medical Center, Department of Emergency Medicine, Jackson, MS; ^ University of Mississippi Medical Center, Department of Otolaryngology, Jackson, MS; ‡ University of Mississippi Medical Center, Simulation and Interprofessional Education Center, Jackson, MS; ** University of Mississippi Medical Center, Department of Biomedical Materials Science, School of Dentistry, Jackson, MS; ^^ University of Mississippi Medical Center, Department of Anesthesiology, Jackson, MS

## Abstract

**Audience:**

This simulator is designed to instruct emergency medicine residents in tracheostomy training that involves bleeding from the tracheostomy site. Any resident, fellow, or attending physician who cares for patients with complications from their tracheostomy might benefit from this innovation.

**Introduction:**

The emergency medicine provider must maintain proficiency in caring for patients with complications from their tracheostomy. In the United States, over 110,000 patients receive tracheostomies per year.^1^ A rare but catastrophic complication of tracheostomies, usually within the first month of placement, is a tracheoinnominate artery fistula (TIAF). This complication occurs in 0.7% of tracheostomy patients and carries a 50–70% mortality.^1^,^2^ We modified a low-fidelity tracheostomy manikin to instruct learners in the stepwise management of hemorrhage from a TIAF.

**Educational Objectives:**

By the end of this educational session, learners will be able to:

1. Perform a focused history and physical exam on any patient who presents with bleeding from the tracheostomy site.
2. Describe the differential diagnosis of bleeding from a tracheostomy site, including a TIAF.
3. Demonstrate the stepwise management of bleeding from a suspected TIAF, including cuff hyperinflation and the Utley Maneuver.
4. Verify that definitive airway control via endotracheal intubation is only feasible in the tracheostomy patient when it is clear, upon history and exam, that the patient can be intubated from above.
5. Demonstrate additional critical actions in the management of a patient with a TIAF, including early consultation with otolaryngology and cardiothoracic surgery as well as emergent blood transfusion and activation of a massive transfusion protocol.

**Educational Methods:**

This modified manikin is a useful training tool for any healthcare provider who is involved in the treatment and stabilization of a variety of tracheostomy emergencies, from bleeding to infection to obstruction or dislodgement. Our case was presented on two separate occasions, to otolaryngology interns (PGY-1), and emergency medicine residents (PGY 1–3). It involved the care of a patient with a sentinel bleed and subsequent hemorrhage from a tracheoinnominate artery fistula (TIAF). This low-fidelity tracheostomy manikin provides the ideal platform for any complex, tracheostomy case, particularly where ongoing bleeding from the tracheostomy site might permanently damage the electrical circuitry of a high-fidelity model. We initially fashioned this modified manikin for tracheostomy training during a simulation “boot camp” for otolaryngology PGY-1 residents. Our use of this modified manikin for tracheostomy training was a useful teaching tool during our otolaryngology intern “boot camp.” As a result, we organized a subsequent simulation training session with our PGY 1–3 emergency medicine residents to provide similar instruction in management of a TIAF.

**Research Methods:**

We provided a pre- and a post-simulation survey for the 33 emergency medicine residents who participated in the TIAF simulation with our modified tracheostomy manikin. There were 11 residents from each of the PGY-1, PGY-2, and PGY-3 year-groups. Thirty-two residents (97%) completed the pre-simulation survey, and 33 residents (100%) completed the post-simulation survey. We used a 6-point Likert Scale from “*strongly agree*” to “*strongly disagree*” to assess a resident’s knowledge of multiple learning objectives within this simulation.

**Results:**

The pre- and post-simulation survey supported this simulation and manikin innovation as a useful teaching tool for tracheostomy emergencies such as a TIAF.

**Discussion:**

This was a useful innovation for emergency provider training in the recognition and management of a TIAF, a rare but emergent tracheostomy complication. In addition to this bleeding complication, this innovation might be useful for a variety of tracheostomy emergencies such as site infection, obstruction, and tube dislodgement. We highly recommend the involvement of both an emergency medicine and otolaryngology content expert in the design and debriefing of tracheostomy cases with this modified manikin. In our experience, a facilitated debriefing by an experienced clinician and educator from both fields provided a diverse perspective for challenging cases such as bleeding from a TIAF.

**Topics:**

Difficult airway, tracheostomy, tracheoinnominate fistula, hemorrhagic shock, tracheostomy complications, Utley Maneuver.

## USER GUIDE

**Table:** 

List of Resources:	
Abstract	1
User Guide	3

**Learner Audience:**

Junior Residents in Emergency Medicine, Senior Residents in Emergency Medicine, Otolaryngology Residents, Surgery Residents, Critical Care Fellows

**Time Required for Implementation:**

It will take approximately 2 hours to create the innovation. Learners will spend approximately 30 minutes using the innovation.

**Recommended Number of Learners per Instructor:**

1–2 instructors: 3–6 learners

**Topics:**

Difficult airway, tracheostomy, tracheoinnominate fistula, hemorrhagic shock, tracheostomy complications, Utley Maneuver.

**Objectives:**

By the end of this simulation learners will be able to:

1. Perform a focused history and physical exam on any patient who presents with bleeding from the tracheostomy site.
2. Describe the differential diagnosis of bleeding from a tracheostomy site, including a TIAF.
3. Demonstrate the stepwise management of bleeding from a suspected TIAF, including cuff hyperinflation and the Utley Maneuver.
4. Verify that definitive airway control via endotracheal intubation is only feasible in the tracheostomy patient when it is clear, upon history and exam, that the patient can be intubated from above.
5. Demonstrate additional critical actions in the management of a patient with a TIAF, including early consultation with otolaryngology and cardiothoracic surgery as well as emergent blood transfusion and activation of a massive transfusion protocol.

### Linked objectives and methods

Emergency medicine providers commonly evaluate patients with complications from their tracheostomy. A focused history and exam are critical elements to determine a potential emergent cause for a tracheostomy bleed (objective 1). This modified tracheostomy manikin allows instructors to present a variety of cases for tracheostomy training, both stable and emergent. A tracheoinnominate artery fistula represents a rare but life-threatening complication from a tracheostomy, usually within the initial month of the procedure. Other emergent complications from a tracheostomy are an acute obstruction, site infection, and most commonly, tube dislodgement. With respect to bleeding complications, it is important for the ED provider to maintain an accurate differential diagnosis, including a TIAF, to properly treat the underlying cause (objective 2).

Shortly after completion of a focused history and physical exam, the simulation operator in this case produces a series of coughs through the overhead speaker. This will cue the nurse (simulated participant) at the bedside to discreetly activate the arterial pump and the patient will begin to hemorrhage from the tracheostomy. At this point in the case, the providers must demonstrate key, stepwise maneuvers to rapidly stop the bleed (objective 3). First, they must provide gentle suction to determine the source of the bleed. As bleeding continues, the providers must then replace the uncuffed tracheostomy tube with a cuffed tracheostomy or endotracheal tube and hyperinflate the cuff. Bleeding temporarily stops but when it restarts, they must slowly pull back the tube and exert anterior pressure on the trachea. Again, bleeding will temporarily stop but when it restarts, the provider must demonstrate the Utley Maneuver. The provider must demonstrate knowledge of each of these steps to prevent ongoing hemorrhage prior to surgical repair. Maintenance of continued pressure via the Utley Maneuver, pending definitive operative repair, is another critical step.

The patient in our case had a tracheostomy after upper airway occlusion from laryngeal cancer. For this reason, our patient would have been unable to have an emergent intubation from above. We modified this manikin to provide occlusion of the larynx, as a means of simulating the laryngeal cancer. If the larynx is unobstructed, then artificial blood will pour from both the tracheostomy and mouth when the pump is turned on. Depending on the underlying indication for the tracheostomy, a patient who might require additional, emergent airway interventions may or may not be able to be intubated from above. This was an important manikin modification and critical information for any provider caring for a patient with a tracheostomy who presents with potential airway compromise (objective 4).

When the patient in this simulation reports “½ cup” of bleeding from the tracheostomy tube, the evaluating provider should be particularly concerned about a tracheoinnominate artery fistula, given that the tracheostomy is only two weeks old. This should trigger the provider to consult early with both an otolaryngologist as well as a cardiothoracic surgeon and consider need for an emergent blood transfusion due to concern for a life-threatening bleed (objective 5). In the absence of uncontrolled bleeding, Interventional Radiology may be another service that can intervene and repair via a stent. Inattention to any of these steps in the simulation might be catastrophic for an actual patient who presents with bleeding from a TIAF.

### Recommended pre-reading for instructor

- Bontempo LJ, Manning SL. Tracheostomy Emergencies. *Emerg Med Clin North Am*. 2019;37(1):109–119. doi:10.1016/j.emc.2018.09.010
- McGrath BA, Bates L, Atkinson D, Moore JA. Multidisciplinary guidelines for the management of tracheostomy and laryngectomy airway emergencies. *Anaesthesia*. 2012;67:1025–41. doi:10.1111/j.1365-2044.2012.07217.x.
- Weingart S. Podcast 195 – Management of Tracheostomy (Trach) and Laryngectomy Emergencies. EMCrit. March 20, 2017. Accessed August 15, 2021. At: https://emcrit.org/emcrit/tracheostomy-emergencies/
- Gupta V, Swaminathan A. CORE EM: Common Tracheostomy Issues. emDOCs.net. June 28, 2019. At: http://www.emdocs.net/core-em-common-tracheostomy-issues/
- Bryant CD. Complications of Airway Devices. In: Tintinalli JE, Ma O, Yealy DM, Meckler GD, Stapczynski J, Cline DM, Thomas SH. eds. *Tintinalli’s Emergency Medicine: A Comprehensive Study Guide*, 9^th^ ed. McGraw-Hill Medical; Accessed March 22, 2021. At: https://accessemergencymedicine.mhmedical.com/content.aspx?bookId=2353&sectionId=221180267

### Learner responsible content (LRC)

- Bontempo LJ, Manning SL. Tracheostomy Emergencies. *Emerg Med Clin North Am*. 2019;37(1):109–119. doi:10.1016/j.emc.2018.09.010
- McGrath BA, Bates L, Atkinson D, Moore JA. Multidisciplinary guidelines for the management of tracheostomy and laryngectomy airway emergencies. *Anaesthesia*. 2012;67:1025–41. doi:10.1111/j.1365-2044.2012.07217.x
- Weingart S. Podcast 195 – Management of Tracheostomy (Trach) and Laryngectomy Emergencies. EMCrit. March 20, 2017. Accessed August 15, 2021. At: https://emcrit.org/emcrit/tracheostomy-emergencies/
- Gupta V, Swaminathan A. CORE EM: Common Tracheostomy Issues. emDOCs.net. June 28, 2019. http://www.emdocs.net/core-em-common-tracheostomy-issues/ Accessed September 10, 2020.
- Bryant CD. Complications of Airway Devices. In: Tintinalli JE, Ma O, Yealy DM, Meckler GD, Stapczynski J, Cline DM, Thomas SH. eds. *Tintinalli’s Emergency Medicine: A Comprehensive Study Guide*. 9^th^ ed. McGraw-Hill Medical; Accessed March 22, 2021. At: https://accessemergencymedicine.mhmedical.com/content.aspx?bookId=2353&sectionId=221180267

### Implementation Methods

Please refer to the simulation submission titled *Tracheoinnominate Artery Fistula* for further details of this case.^3^

- Video of how to create this innovation: https://youtu.be/hbxC8D9D1bQ
- Tarver E M, et al. Tracheoinnominate Artery Fistula. JETem 2021. 6(3):S62–86. https://doi.org/10.21980/J8K05R

### List of items required to replicate this innovation

1. **Laerdal NG Tube and Trach Care Trainer** https://laerdal.com/products/skills-proficiency/airway-management-trainers/ng-tube-and-trach-care-trainer/
2. **Arterial Line Pump and Tubing** *hand pump version:* https://www.acehardware.com/departments/lawn-and-garden/outdoor-powerequipment/funnels/8135238?store=16748 (*IV Pumps or other motorized peristaltic pumps work well and vary in cost)*
3. **Artificial Blood**
*Alkaline water is readily available at most large retail stores*
https://www.walmart.com/ip/Alkaline-Water-with-Himalayan-Minerals-Electrolytes-1-Gallon/36357542?wl13=875&selectedSellerId=0
*Phenolphthalein 1% Indicator Solution can be purchased online*
https://www.homesciencetools.com/phenolphthalein-solution-30-ml/
4. **Synthetic caulk to plug oropharynx of task trainer** https://www.amazon.com/Gorilla-Percent-Silicone-Sealant-Squeeze/dp/B01B5RBOA6/ref=sr_1_5?dchild=1&keywords=synthetic+caulk&qid=1618159604&s=hi&sr=1-5
5. **LocTite Super Glue** https://www.amazon.com/Loctite-Ultra-Control-4-Gram-1363589/dp/B003Y49R7G/ref=asc_df_B003Y49R7G/?tag=hyprod-20&linkCode=df0&hvadid=192269250190&hvpos=&hvnetw=g&hvrand=11043970704346008128&hvpone=&hvptwo=&hvqmt=&hvdev=c&hvdvcmdl=&hvlocint=&hvlocphy=9060534&hvtargid=pla-308428871170&psc=1
6. **A 6.5 Endotracheal tube adaptor to connect pump tubing to the right mainstem bronchus**
7. **Umbilical cord clamp or similar fastener to occlude the left mainstem bronchus**

### Approximate cost of items to create this innovation

1. **Laerdal NG tube and Trach Care Trainer:** New model costs approximately $1500.00. Many established simulation centers may have these already on-hand. (This modification is reversible if the simulation staff would like to convert back to the original trainer after the exercise.)
2. **Arterial pump with tubing**: This is needed for connection to the right mainstem bronchus of the trainer. We used the Gaumard Arterial Pump which was discontinued several years ago. However, any peristaltic pump or even a hand pump would provide a reasonable substitute. Cost will vary according to the type of pump. A hand pump will cost as little as $6.00 from the local hardware store.
3. **Artificial Blood:** The cost of alkaline water is approximately $4.00, and the cost of 1% phenolphthalein indicator solution is approximately $7.00. Our artificial blood was useful because it would turn clear after hitting the towel, or manikin surface but appeared dark red when pouring from the tracheostomy tube. As a result, it does not permanently stain the manikin or other materials and allows for quick turn-over between repeat cases.
4. **Synthetic material to plug the upper airway of the manikin:** A member of our dental school faculty used additional-reaction silicone elastomer dental material to plug the larynx of this manikin so that he could not be intubated from above. This material is also known as polyvinyl siloxane (PVS) impression material. It is widely used in dental practice to register the form and relation of the teeth and the surrounding tissue. It has also been useful in other non-dental-related fields such as fractography for replicating fracture surfaces because of its high definition, long-term dimensional stability, low dimensional change, relatively short setting time, and high tear resistance. Other advantages of this material are the different consistencies (low, medium, heavy, and putty) and shades available. We used the transparent low viscosity (Elite Transparent, Zhermack) and light brown silicone (Extrude Light bodied, Kerr) available in a 50 ml cartridge that is placed in a dispensing gun to extrude the impression material. Mixing tips (~70 mm long) are used for mixing the material per package instructions and for easier application. Silicone caulk could provide a similar, water-tight substitute and costs between $5.00 and $10.00 from any hardware store.
5. **LocTite Super Glue:** There are two layers of plastic at the tracheostomy stoma. We slightly enlarged this orifice to accommodate the Utley Maneuver in this exercise and used LocTite Super Glue to adhere these layers back together so that artificial blood from the simulation would not track between them. This glue costs approximately $5.00 at most retail stores.

### Detailed methods to construct this innovation

1. Remove the chest flap of the NG Tube/Trach Care Trainer and manually remove the inflatable bag (simulated right lung) from the right mainstem bronchus. This should detach without excessive force. Attach exit tubing from the pump apparatus to this bronchus. We used a 6.5 endotracheal tube adaptor to connect the right bronchus to the pump tubing. Occlude the left mainstem bronchus with an umbilical cord clamp or similar device.
  **Figure:**
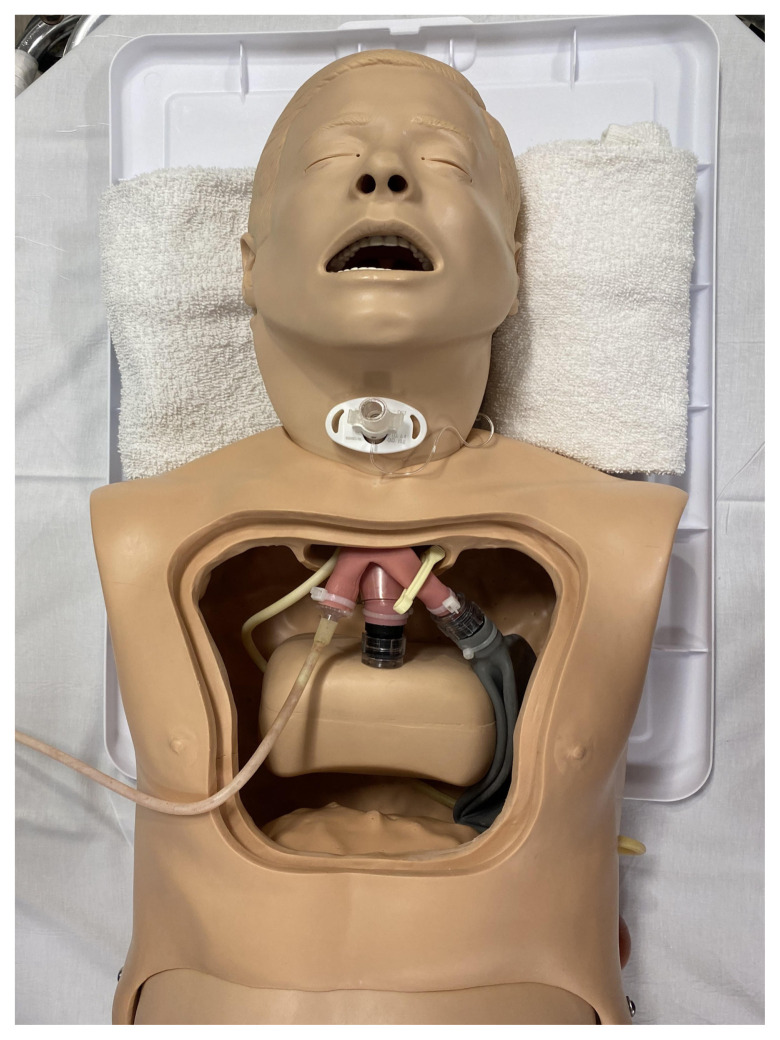
Internal Modifications to Low-Fidelity Manikin for TIAF Simulation
2. This step is optional but you may consider slightly enlarging the tracheostomy stoma size to accommodate both the endotracheal tube and gloved finger for the Utley Maneuver. If this is done, use superglue to re-adhere the two layers of plastic at the tracheostomy site so that there is no remaining gap between these layers.
  **Figure:**
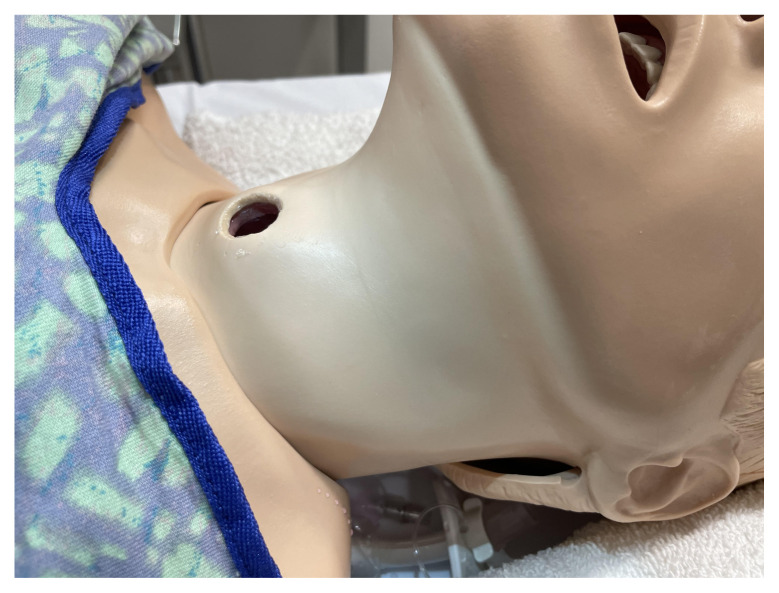
Superglue is applied to reinforce the two layers of the manikin at the tracheostomy

**Figure:**
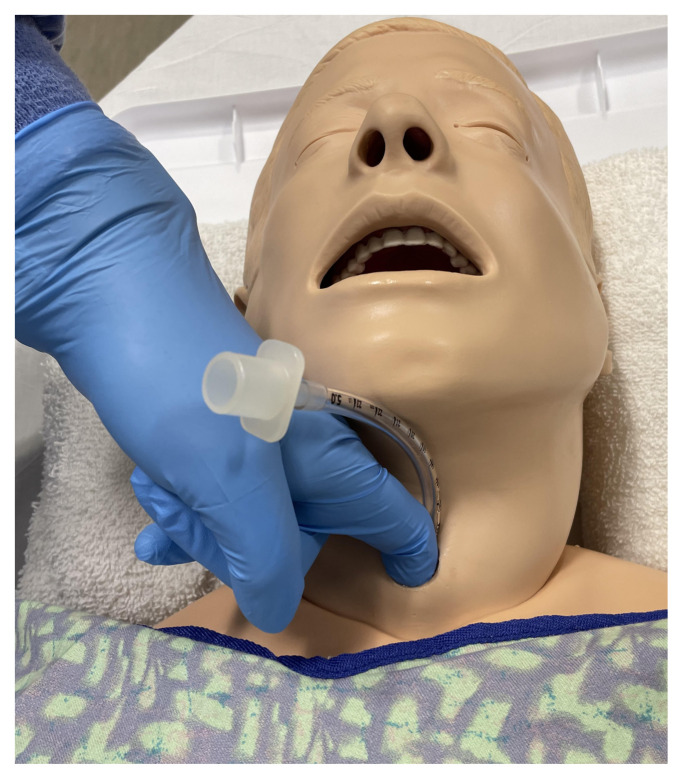
Utley Maneuver
3. Use a video or direct laryngoscope to identify the larynx. Occlude the larynx at the vocal cords with a synthetic caulk.
  **Figure:**
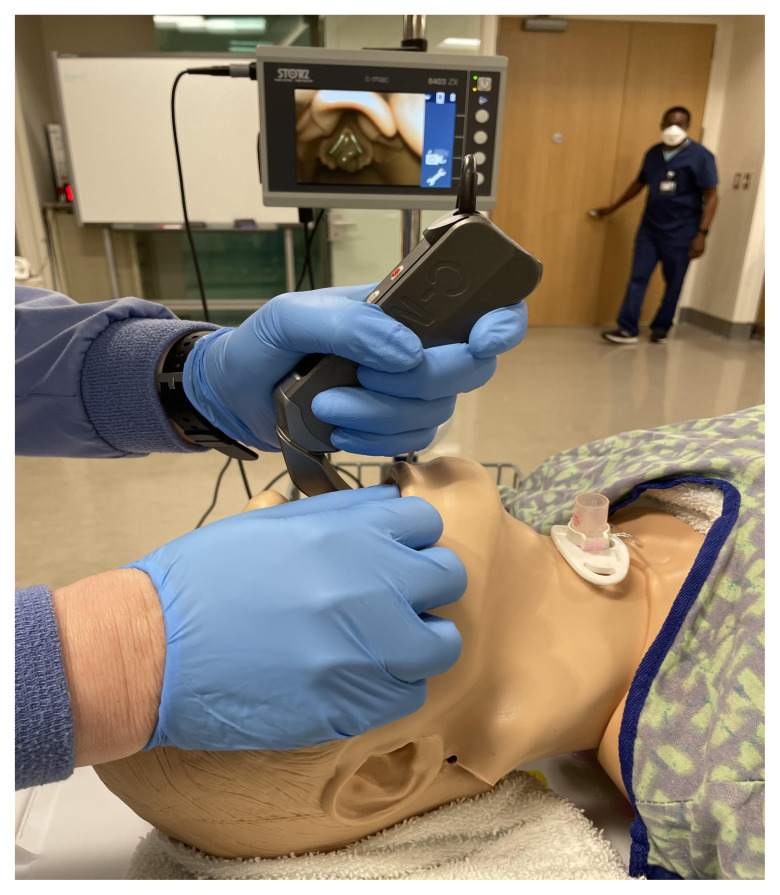
Video Laryngoscope to View Occlusion of Manikin at Larynx

**Figure:**
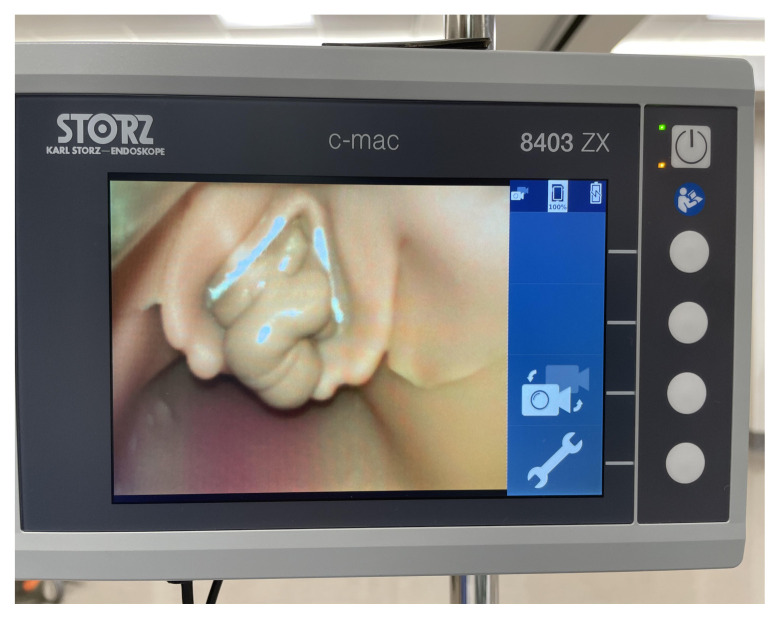
Additional View of Larynx of Modified Manikin
4. Create artificial blood with 1 gallon of alkaline water (pH 9); add enough drops of 1% phenolphthalein solution to turn the water a dark magenta color. This artificial blood will appear dark magenta when it is pumped out of the tracheostomy site but will quickly turn clear upon striking the surface of the manikin, towel, or floor. Place the gallon of artificial blood on a stand to the side of the patient. Connect the pre-pump tubing from the gallon to the pump. The post-pump tubing should already be connected to the mainstem bronchus of the manikin.
  **Figure:**
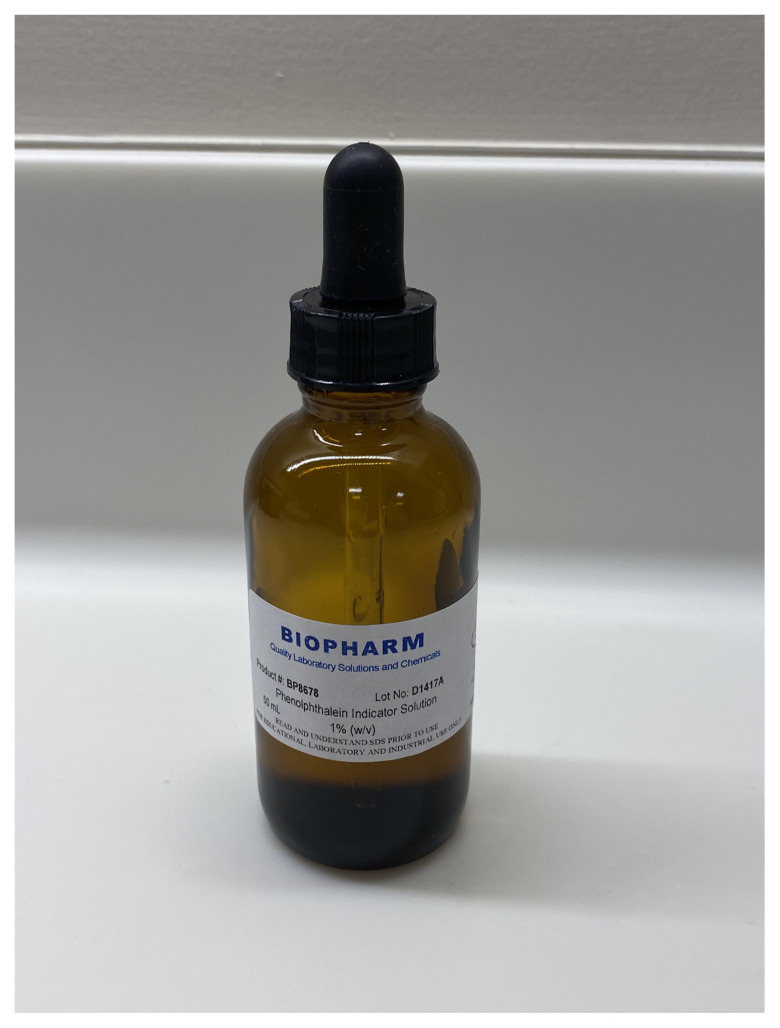
Phenolphthalein 1% Indicator Solution to Use to Create Artificial Blood

**Figure:**
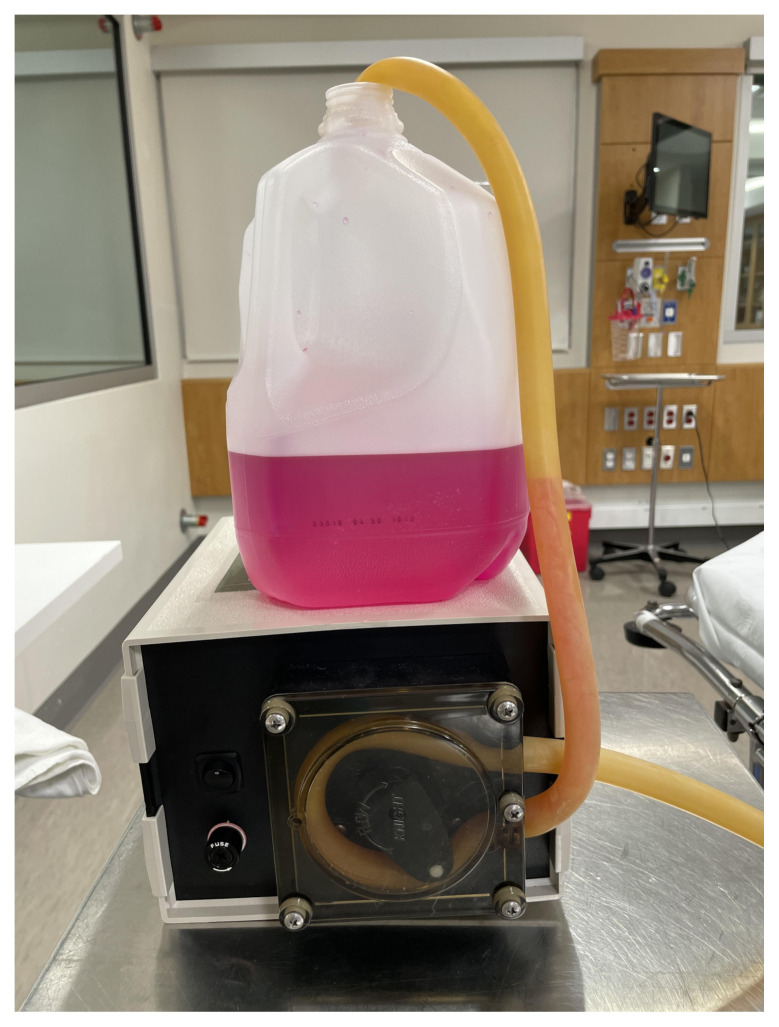
Artificial blood with pump and tubing

**Figure:**
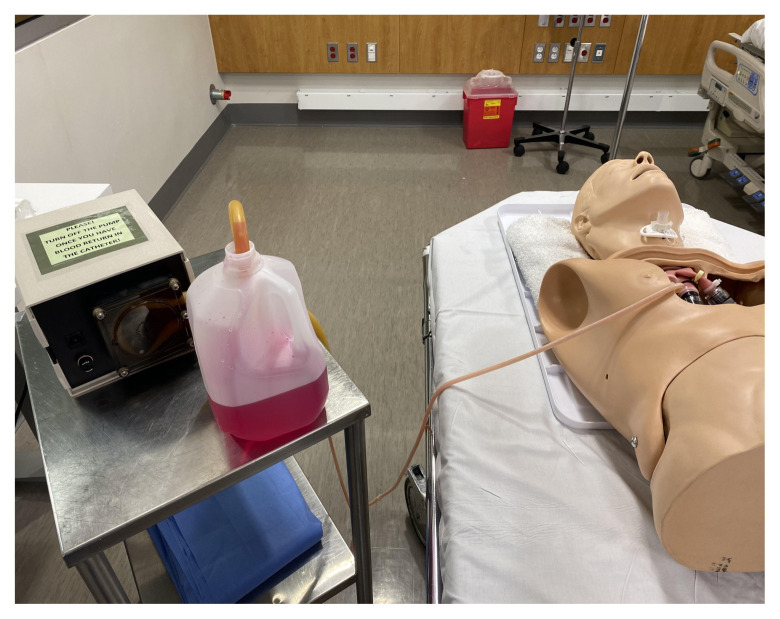
Open Set-Up with Artificial Blood and Modified Manikin
5. Cover the pump, artificial blood, and tubing with a sheet or towel during the case. Cover the manikin with a gown. It is best to keep the chest flap off the manikin so that it does not compress the pump tubing. Place a towel under the head and neck of the manikin to absorb the fake blood upon activation of the pump.
  **Figure:**
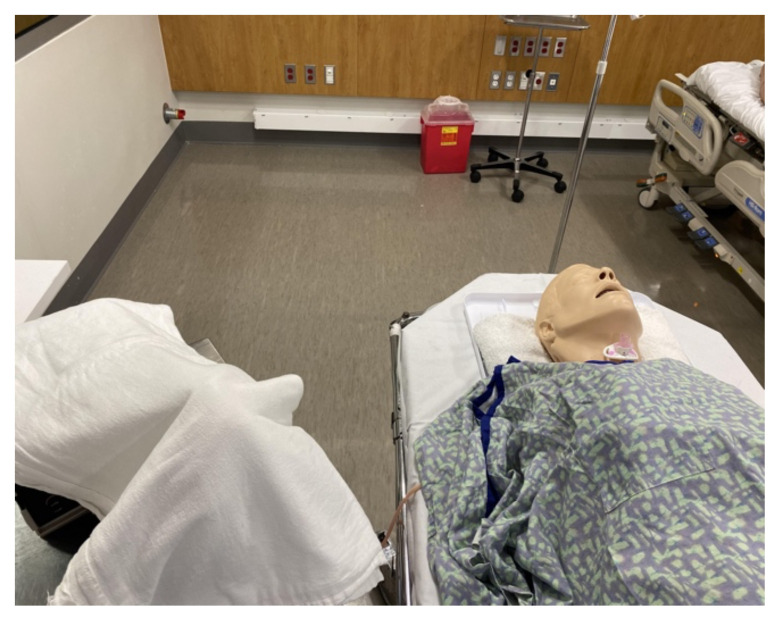
Manikin and Covered Pump Before Simulation
6. Place a size 6 uncuffed tracheostomy tube in the tracheostomy site. Provide an airway cart with a similarly-sized, cuffed tracheostomy tube as well as a smaller (size 5), uncuffed endotracheal tube. If modified correctly, artificial blood will pour primarily from the tracheostomy tube during the simulation.
  **Figure:**
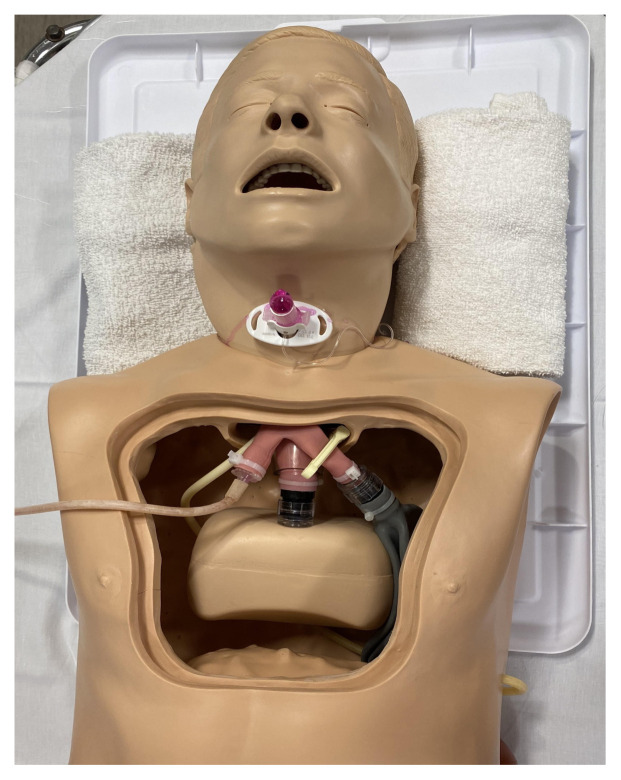
Hemorrhage from Tracheostomy Tube

### Results and tips for successful implementation

This innovation is best implemented in a simulation center with facilitation by content experts in both emergency medicine and otolaryngology. We used this innovation in a simulation which was completed with 33 junior- and senior-level emergency medicine residents. The research was considered exempt by the Institutional Review Board at our institution. A pre- and post-simulation survey strongly supported the use of this innovation in the emergent management of a TIAF. Additional learners who might benefit from this simulation include otolaryngology and general surgery residents as well as critical care fellows, and attending physicians in each of these specialties. In set-up for this simulation, it is helpful to place the pump on a mayo stand beside the manikin. We used a peristaltic pump, but a hand pump would be a low-cost alternative. We covered the pump with a sheet and connected the tubing to the right mainstem bronchus of the manikin. The simulated participant, playing the nurse, was positioned between the patient and the mayo stand to conceal this apparatus from the learners. Throughout the TIAF case, our simulation operator coughed several times through the microphone to cue the nurse to turn on the pump. We involved another simulated participant during the case who acted as a family member to provide additional history. This additional interaction assisted the learners in obtaining necessary information at the start of the case, due to the patient’s critical illness and inability to speak. We initially offered a total of 40 minutes for use of the innovation, simulation, and debriefing. This was too short for adequate time in the debriefing, and we would modify this in the future to a 60-minute activity. It is important to allow ample time in debriefing for discussion on management of a tracheoinnominate artery fistula as well as general teaching on the emergent management of tracheostomy complications.

## Supplementary Information


